# Oral and Long-acting Injectable Aripiprazole in Severe Mental Illness and Substance Use Disorder Comorbidity: An Updated Systematic Review

**DOI:** 10.2174/1570159X23666241023115252

**Published:** 2024-10-24

**Authors:** Mario Santorelli, Andrea Miuli, Mauro Pettorruso, Francesco Di Carlo, Domenico De Berardis, Stefano L. Sensi, Giovanni Martinotti, Massimo Clerici, Massimo di Giannantonio

**Affiliations:** 1 School of Medicine and Surgery, University of Milano Bicocca, Monza 20900, Italy;; 2 Department of Neuroscience, Imaging, and Clinical Sciences, University G. d'Annunzio of Chieti-Pescara, Chieti, Italy;; 3 Department of Mental Health, ASL Lanciano-Vasto-Chieti, Chieti, Italy;; 4 National Health Service, Department of Mental Health, ASL 4, Teramo 64100, Italy;; 5 Molecular Neurology Unit, Center for Advanced Studies and Technology - CAST, Institute for Advanced Biomedical Technology-ITAB University G. d'Annunzio, Italy;; 6 Department of Surgery and Interdisciplinary Medicine, University of Milano-Bicocca, Monza 20900, Italy;; 7 Department of Mental Health, Azienda Ospedaliera San Gerardo, Monza 20900, Italy

**Keywords:** Schizophrenia, bipolar disorder, dual disorder, substance use disorder, aripiprazole, craving

## Abstract

**Background:**

Co-occurrence of substance use disorders is frequent in patients with mental health disorders is a condition known as “dual diagnosis”. The use of substances worsens the prognosis and lowers the quality of life of psychiatric patients. It also increases the risk of hospitalization and suicide rate.

**Objectives:**

To assess the effects of aripiprazole therapy on substance use and other psychiatric outcomes in dually diagnosed patients.

**Methods:**

We performed a systematic review conducted on 3 databases PUBMED, SCOPUS, and Web of Science, selecting original studies and analyzing the impact of aripiprazole therapy on dually diagnosed patients. Six hundred and fifty-five articles were founded and, after removing duplicates (n = 274) and applying the exclusion criteria, 12 articles were included in our systematic review.

**Results:**

12 studies were included, among which 6 were Randomized Controlled Trials. The Most frequent psychiatric diagnosis were schizoaffective disorders, schizophrenia, and bipolar disorders. Alcohol and cocaine use disorders were the most used substances. Eleven studies showed a clinical improvement after aripiprazole treatment. 8 studies evaluated craving and found a significant reduction after treatment with aripiprazole. No definitive conclusions can be drawn on substance usage and maintenance of abstinence.

**Conclusion:**

The present findings suggest aripiprazole may be associated with reducing substance craving and improving depression, psychosis, and schizoaffective disorders in dually diagnosed patients.

## 1 BACKGROUND

The co-occurrence of a Substance Use Disorder (SUD) is more frequent in patients with mental disorders compared to the non-psychiatric population (*e.g*., 47% of patients with schizophrenia have severe problems with drugs or alcohol during their lifetime compared to 16% of the general population) [1]. The co-occurrence of SUD and psychiatric diagnosis is a new nosological entity known as dual diagnosis [2]. The presence of SUD worsens the prognosis and quality of life of psychiatric patients [3], thereby increasing clinical exacerbations, hospitalization, poor compliance with treatment, and suicide rates [3-5]. The high prevalence of SUD in psychiatric patients has been explained by 3 models: the self-medication model, the sharing of risk model, and the substance-induced model [6]. In the first one, substance use is the favorite viaticum for avoiding the anguish of the illness or simply the easiest way to find a reward. According to the sharing of risk model, the correlation between psychiatric disorders and SUD relates to sharing the same risk factors. The “substance-induced model” postulates that a patient develops another psychiatric diagnosis after a brief or prolonged use of substances. The biological bases of the correlation between SUD and primary psychiatric diagnoses like schizophrenia, schizoaffective disorder, and bipolar disorder involve the abnormal activation of the mesolimbic dopamine system [7-10].

Antipsychotics, through the activation of dopaminergic mesocortical and mesolimbic pathways, affect substance use [11]. Atypical antipsychotics are preferred for the lowest incidence of extrapyramidal side effects and as they promote the stabilization of dopaminergic neurotransmission in reward systems [12]. Aripiprazole is an atypical antipsychotic characterized by partial agonist activity at dopamine D2, D3, and serotonin 5-HT_1A_ receptors [13]; the compound also has antagonist activity on serotonin 5-HT_2A_ receptors [14]. This plethora of effects contribute to reducing positive, negative, and cognitive symptoms of schizophrenia [15] and favors mood stabilization in bipolar disorder [16].

In treating dual-diagnosis patients, aripiprazole is used to reduce substance use and manage the symptoms related to the addiction likewise craving, tolerance and abstinence [17]. Craving has a pivotal role in the perpetuation of drug use [18], representing one of the key symptoms of SUD [19, 20]. It could be described as an intense desire for the substance, a condition induced by the urgency for a new assumption [21]. The craving state is linked with a mesolimbic and glutamatergic neuroadaptation induced by drugs or behaviors and consists of decreased cognitive control and enhanced glutamateric drive in response to potential drug-associated rewards [22]. Furthermore, craving serves as the major motivator to propagate drug use and to elicit relapse in abstinent individuals [23]. The continuous use of addictive drugs.

Through the repeated potentiation of the mesolimbic dopamine (DA) system produces potent and long-lasting changes in the overall dynamics of neural circuits that control motivated behaviors [24]. The partial agonism of aripiprazole on the D2 and D3 receptor, working as “stabilizator” of dopaminergic synapsis, could impact the drug-seeking by both reducing the craving and indirectly the intake of substances [25].

This study examines the efficacy of aripiprazole in treating dually diagnosed patients and evaluates substance abstinence, craving, and effects on other psychiatric diagnoses.

## 2 MATERIALS AND METHODS

Our systematic review was conducted to study the potential role of aripiprazole in a population of patients with dual diagnosis. We performed a survey on PubMed, Scopus and Web of Science (WoS) on May 15, 2021 using the following search strategy: Aripiprazole and (“dual diagnosis” or “comorbid substance use” or alcohol or cocaine or opioid or cannabis or hallucinogens or amphetamines) NOT review. Exclusion criteria for the first and the second phase of screening were: (1) Non-original research (*e.g*., review, commentary, editorial, book chapter, case series, case report); (2) Non-full-text article (*e.g*., meeting abstract); (3) language other than English; (4) Animal/*in vitro* studies; (5) Non-Dual Diagnosis; (6) No data on efficacy. We found 655 articles (PubMed = 247; Scopus = 156; WoS = 252). After removing duplicates (N = 274), we screened 381 records. 51 were non-original (letter to editor without data available, review, meta-analysis, commentary, editorial, book chapter); 10 were non-full-text articles, 10 were not written in English, 295 were not related to the focus of the review (animal/*in vitro* studies = 115, non-dual diagnosis = 164, not on aripiprazole = 16) and three studies had no data on efficacy. Twelve articles were eventually taken into consideration for qualitative synthesis. The method is summarized in Fig. (**1**).

## 3 RESULTS

In our systematic review, we evaluated a total of 12 studies. All the characteristics of the included articles are described in Table **1**. Among the included studies, 6 were Randomized Controlled Trials (RCT) [26-31] and 6 were observational studies; a total of 283 dual-diagnosed patients (56 female and 227 male) were treated with aripiprazole; 160 out of 283 (23 female and 137 male) were enrolled in the RCTs. Two hundred and seven subjects (28 female and 179 male) were recruited as a control group; only 15 (4 female and 11 male) were healthy control. The psychiatric pathologies treated concurrently with the SUD were: schizoaffective disorder (N = 5 [26, 32-35], followed by schizophrenia (N = 4 [26, 32, 34, 36]), bipolar disorder (N = 4 [27, 34, 35, 37]), schizophrenia spectrum (N = 2 [27, 30]), personality disorder [37], substances-induced psychosis [28], history of psychotic symptoms [30] and major depressive disorder [29] (N = 1 studied each). Eleven studies [27-37] showed a clinical improvement after the aripiprazole treatment. In particular, three studies [27, 36, 37] highlighted the significant improvement produced by aripiprazole in the quality of life of dually diagnosed patients. The most investigated substances were cocaine in eight studies [26, 27, 31, 32, 34-36] or alcohol in eight studies [27, 31, 32, 34-38]. Eight studies evaluated craving, and all of them found a significant reduction in craving after treatment with aripiprazole [26, 27, 30-32, 35, 37, 38]. There are no clear results regarding the quantity of substance use and maintenance of abstinence. Two studies did not find a significant effect on reducing the amount of substance or maintaining abstinence after aripiprazole therapy [26, 29]. In contrast, significant positive results were found in other studies that indicated a reduction of positive urine dosage [32], reduction of the quantity of substance used [39], reduction in money spent on substances [35, 39], reduction of withdrawal symptoms [37], and reduction of the severity of addiction [36]. Only one open-label study showed the effects of aripiprazole plus topiramate in dually diagnosed patients after methadone withdrawal. This approach significantly reduced the Positive and Negative Symptom Scale (PANSS) and Brief Psychiatric Rating Scale (BPRS), and maintained abstinence in all the patients [33]. Only four studies [26-28, 31] compared aripiprazole to other typical [26, 31] and atypical antipsychotics [27, 28, 31]. Aripiprazole is better than typical (haloperidol and perphenazine [26, 31]) and atypical antipsychotics (paliperidone and quetiapine [27, 31]) in reducing craving. Two studies evaluated the efficacy of long-acting aripiprazole and indicated a significant reduction in craving [27, 36].

### 3.1 RCT Studies in Deep Analysis

The group of Beresford *et al.* compared aripiprazole 10-30 mg with perphenazine (dosage 12-24 mg), reporting a significant reduction in craving intensity and duration without substantial changes in the maintenance of abstinence-only in the aripiprazole group [26]. In another study comparing aripiprazole 10-20 mg/die *versus* haloperidol 15-30 mg/die, similar results on craving were obtained with significant reduction of craving and reduction on PANSS and BPRS [31]. In the same study, aripiprazole showed a significant reduction in both craving and PANSS but not in BPRS when compared to quetiapine 300-600 mg/die. Furthermore, Sulaiman *et al.* compared aripiprazole 5-10 mg/die *versus* placebo in patients with methamphetamine dependence and a history of psychosis [30]. The aripiprazole group was significantly better in craving reduction and substantially improved Clinical Global Impression (CGI) and PANSS but did not perform better in maintaining abstinence. One study used aripiprazole 5-15 mg in combination with escitalopram 10-20 mg/die in patients with depression and alcohol use disorder [29]. The escitalopram plus aripiprazole groups performed significantly better in craving reduction than escitalopram plus placebo. Both groups did not differ in maintaining abstinence or the Back Depression Inventory Scale (BDI) and CGI subscales. Moreover, no significant results were found in the Scale for Assessment of Positive Symptoms (SAPS) and Scale for Assessment of Negative Symptoms (SANS) when comparing aripiprazole 15 mg *vs.* risperidone 2-4 mg [28]. Cuomo *et al.* analyzed the effectiveness of long-acting aripiprazole therapy (300/400 mg/4 weeks) *versus* long-acting paliperidone (100 mg/4 weeks). Aripiprazole performed significantly better in craving reduction and on quality-of-life scales [27].

### 3.2 Safety and Tolerability

Aripiprazole presented a good profile of safety and tolerability in all the included studies. No studies reported significant changes in basal parameters or the onset of acute extrapyramidal effect, seizures, or cardiac events. The most common side effects are mild to moderate. Nausea and vomiting are the most frequent side effect reported [32, 37, 38] and lead in rare cases to treatment discontinuation. Insomnia [33, 35] and headache [31, 38] are other frequent adverse event followed by movement disorders such as tremor, agitation or akathisia [27, 30].

## 4 DISCUSSION

Alterations of dopaminergic neuro transmission increased striatal-limbic activation and disrupted prefrontal control are considered the neurobiological signatures that predict drug craving and drug use [40]. In our systematic review, we found a significant reduction of craving with aripiprazole treatment in the dually diagnosed patient. The compound produced better results when compared to first- (haloperidol, perphenazine) [26, 31] or second-generation (quetiapine, paliperidone) antipsychotics [27, 31]. The efficacy of aripiprazole is at least partially explained by its partial agonism of the DRD2 and DRD3 dopamine receptors. The dopamine D1, D2, and D3 receptors play a crucial role in seeking substances and maintaining addiction. Low-affinity D1 receptors, which stimulate cyclic adenosine monophosphate (cAMP) signaling, are involved in acute drug reward and maintaining motivation to take drugs [41]. In contrast, D2 receptors and the inhibition of cAMP signaling are stimulated by phasic and tonic dopaminergic activity. D2 receptor activation may counteract drug reward [42]. The different polymorphisms of the DRD2 gene seem to play an important role in the sensitization of D2 receptors to aripiprazole, in particular, DRD2/ ANKK1 Taq1A seems to be linked to the efficacy and development of side effects of the drug [43]. Furthermore, high-affinity D3 receptors are associated with drug-seeking behavior [44]. The DRD3 signaling in the ventral putamen is also a critical and specific mediator of relapse to cocaine-seeking after prolonged abstinence [25]. For this reason, the strong effect on craving reduction of aripiprazole can be explained by its modulation of the DRD3-dependent pathways involved in substance seeking. Another important finding is the significant reduction of craving after implementing aripiprazole on antidepressant therapy in major depressive disorder (MDD) patients with SUD [29]. This suggests that targeting dopaminergic pathways in MDD improves the disease outcome. The effect of aripiprazole on craving remains significant even when changing the substance used. Although alcohol and cocaine have completely different actions (Alcohol is a sedative while cocaine has stimulant effects), aripiprazole reduces craving in both additions. This result supports the notion that similar pathways are involved in craving regardless of the type of substance used.

As reported in several studies, another aspect to consider is the possibility that aripiprazole (pramipexole and ropinirole) may increase the risk of developing pathological gambling [45]. This behavioral addiction (often called “addiction without substance”) seems to share several aspects with the SUD. For this reason, in the past, a primary alteration of the DRD2 system has been attributed to the development of pathological gambling, just like in the SUD. In a recent systematic review of the literature [46], it would seem that the development of gambling behaviors has a complex genesis and cannot be explained only by an alteration of DRD2. The absence, in our systematic review, of an increased risk of drug intake following aripiprazole administration could be explained by these different and not well-investigated neurobiological differences between gambling and SUD.

One of the main concerns in dual-diagnosis patients is the low rate of treatment compliance [47]. Long-acting (LAI) antipsychotics are used as maintenance therapy in chronic schizophrenia and are associated with a significantly lower risk of hospitalization or relapse than oral antipsychotics as they significantly improve compliance to therapy [48]. Similarly, long-acting treatment on dually diagnosed patients reduces relapse rate and increases adherence to therapy [49]. This result was confirmed when evaluating long-acting aripiprazole, a therapy that we found produced positive results, particularly on craving reduction and improvement of psychometric parameters [27, 36]. LAI therapy can maximize the positive effects of aripiprazole on substance seeking and promote better control of other psychiatric symptoms.

## 5 LIMITATIONS AND FUTURE PROSPECTIVE

The main limitation of this study is the high heterogeneity of the included studies in terms of drug dosage and administration, psychometric assessment, substances used, control group and the few RCT. Due to the lack of homogeneity between studies the author decided to not add a meta-analysis to this systematic review. According to the results we found there is not a clear association between the use of aripiprazole and the maintenance of abstinence. Consistently, more studies are needed to better clarify the link between the reduction of craving and substance consumption. Finally, another possible limitation is the absence of a registered protocol for the review.

## CONCLUSION

Despite the growing literature on the treatment of dually diagnosed patients, the current knowledge about the clinical effects of antipsychotic therapy is still limited. The present findings suggest that aripiprazole is associated with a reduction in craving for substances and improvement of primarily diagnosed illnesses such as depression, psychosis, and schizoaffective disorders. A greater number of large-population RCTs and more homogeneous quantification of cravings are nevertheless needed to confirm our findings.

## Figures and Tables

**Fig. (1):**
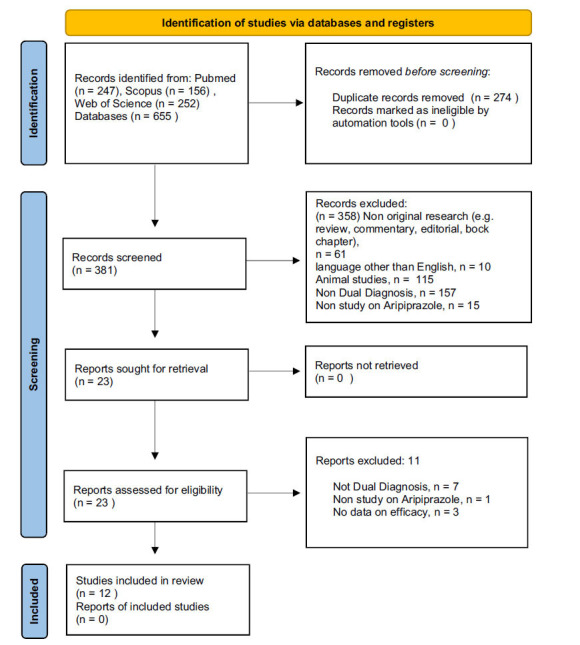
PRISMA 2020 flow diagram for new systematic reviews which included searches of databases and registers only. BMJ (OPEN ACCESS) Page MJ, McKenzie JE, Bossuyt PM, Boutron I, Hoffmann TC, Mulrow CD, *et al*. The PRISMA 2020 statement: an updated guideline for reporting systematic reviews. BMJ 2021;372:n71. doi: 10.1136/bmj.n71.

**Table 1:** Summary of the main results.

**References**	**Population (N) Total, Male l (N_m_), Diagnosis**	**Treatment Assigned (Drug, Dosage)**	**Measures**	**Substances Use:** **Main Results**	**Psychopathology:** **Main Results**
Beresford *et al.*, 2005 [32] Open-label	N = 10, N*_m_* = 10 Schizophrenia or schizoaffective disorder (DSM-4) Cocaine dependence and Alcohol abuse (DSM-4)	Aripiprazole 10-15 mg/day. No concurrent medications are allowed.	Urine drug screens (UDS); TLFB; BSCS for cocaine and alcohol; BPRS	Positive USD dropped significantly. Weekly TLFB scores dropped significantly which means BSCS declined significantly both for cocaine and alcohol.	BPRS scores lowered significantly from baseline to endpoint. Significant reduction of cocaine craving BSCS and psychosis BPRS. Significant Association in reduction of alcohol craving BSCS and psychosis BPRS.
Bruno *et al.*, 2014 [33] Open Label	N = 20, *N_m_* = 10 Opioid dependence DSM-4 schizoaffective disorder DSM-4	Aripiprazole 10 mg/day. Topiramate increased from 50 mg/day to 200 mg/day at week 4 (100 mg/day twice daily with increments of 50 mg/day mg/week). Methadone Starting dose 80 mg/day. Decrease 20 mg per week.	PANSS; BPRS; HAM-D, HAM-A; DOTES	Not abstinence symptoms post methadone suspension	PANSS significantly dropped. BPRS significantly reduced significant reduction in HRSD and HAM-A
McRae-Clark *et al.*, 2009 [34] Open-label	N = 20, N*_m_* = 17; Schizophrenia, Schizoaffective Disorder, or Bipolar Disorder DSM 4 Alcohol, Cocaine or Marijuana abuse or dependence DSM 4	Aripiprazole: from 15 mg/day up to 30 mg/day.	Blood and urine drug test, AIMS, PANSS MADRS/YMTM	Cannabis: ns difference. Cocaine: significant reduction of money spent <0.01 but Ns reduction of cocaine use. Alcohol: significant reduction in Alcohol use.	PANSS significant reduction in Schizophrenia and Schizoaffective Disorders. MADRS and YMRS ns. CGI ns reduction in Bipolar and Schizophrenia and Schizoaffective Disorders.
Brown *et al.*, 2005 [35] Open Label	N = 20, *N_m_* = 11 Bipolar I, II, not otherwise specified, Schizoaffective disorders (DSM-4). Abuse/dependence on cocaine, amphetamines, cannabis, opiates, or alcohol (DSM-4).	Aripiprazole 30 mg/day.	UDS, VAScrav, days of use and dollars spent on substances in the past week, HAM-D; YMRS; AIMS; MINI SAS; BAS	In alcohol-dependence patients (N = 17), significant reductions of VAScrav and reduction in dollars spent/week on alcohol. In cocaine-dependence patients, significant reduction in VAScrav.	baseline-to-exit significative improvement on the HAM-D, YMRS, and BPRS.
Szerman *et al.*, 2020 [36] Open-label	N = 40, N*_m_* = 31 Substance use disorder DSM 5 Schizophrenia DSM 5	Aripiprazole 400 mg/4 weeks; 300 mg/4 weeks.	CGI, WHODAS-2.0, SDS	Significant reduction of SDS in alcohol use disorders and cocaine use disorders.	WHODAS 2.0 significant reduction at both 3- and 6-month *vs.* baseline. CGI significant reduction at 3- and 6-month *vs.* baseline.
Martinotti *et al.*, 2007 [37] Open-label	N = 13, N*_m_* = 11; Personality disorders, Bipolar disorders, DSM 4 Alcohol dependence, cannabis dependence cocaine dependence DSM 4	Aripiprazole 5-15 mg/day.	CGI, SCID I and II, EuropASI, CIWA-Ar, VAScraving, OCDS., SCL-90 R, QOL index	Withdrawal symptoms reduction, VAScrav significant reduction.	Significant improvement of CGI and QOL. OCDS: significant reduction of compulsive subscale. SCL-90 R General severity significant reduction.
Beresford *et al.*, 2017 [26] RCT	N = 44, *N_m_* = 33 Group 1 N = 22, *N_m_* = 16 Group 2 N = 22, *N_m_* = 15 Schizophrenia or schizoaffective disorder (DSM-4) Cocaine dependence (ICD-10)	Group 1 = Aripiprazole 10-30 mg/once a day + 2 placebo doses. Group 2 = Perphenazine 4 to 8 mg/3 times a day.	UDS; BSCS; PANSS; CGI; TLFDA; MINI; MSES	UDS ns, compliance difference ns, craving intensity between group: ns; arm-by-time analysis: significant decreases in Group 1 for both duration and frequency of craving. Comparing weeks 3-5 to 6-8 in Group 1 significant reduction in craving intensity, frequency and duration. Ns carving reduction in Group 2.	No data reported
Cuomo *et al.*, 2018 [27] RCT	N = 101, *N_m_* = 81 Group 1 N = 50, *N_m_* = 41 Group 2 N = 51, *N_m_* = 40 Schizophrenia spectrum and other psychotic disorders or bipolar disorder with psychotic features comorbid with substance use disorders DSM 5	Group 1: Aripiprazole Maintena 400 mg / monthly + 10-20 mg/day aripiprazole for 2 weeks. Group 2: Paliperidone Palmitate 150 week 1 +100 mg week 2; 100 mg/monthly.	VAScrav; WHODAS-BREEF CGI; BPRS	VAScrav significant reduction in groups 1 and 2. Group 1 was significantly better in VAScrav *vs.* group 2.	CGI improved significantly in both group. Group 1: Significant improvement WHOQOL-BREF all domains. Group 2: Significant improvement WHOQOL-BREF all domains. Group 1 significantly better WHOQOL-BREEF *vs*. Group 2.
Farnia *et al.*, 2014 [28] RCT	No = 45, *N_m_* = 45 Group 1, No = 22, *N_m_* = 22 Group 2, No = 23, *N_m_* = 23 Methamphetamine dependence DSM 4 Substances-induced psychosis DSM-4	Group 1: aripiprazole 15 mg/day. Group 2: risperidone 4 mg/day.	SAPS, SANS	Not evaluated.	Significant reduction of SAPS in both group. SANS: Reduction in both groups but NS *p* = 0.08.
Han *et al.*, 2017 [29] RCT	No = 50, N*_m_* = 44; Group 1 No = 17, N*_m_* = 10 Completed trial No = 14, N*_m_* = 9 Group 2 N = 18, N*_m_* = 13 Completed trial N = 17 N*_m_* = 12 Group 3 N = 15, N*_m_* = 11 Mayor depressive disorder and Alcohol dependence with DSM 4	Group 1: escitalopram (10 mg/day starting dose, after a week 20 mg/day) + aripiprazole (5 mg/day starting dose up to 15mg/day). Group 2: escitalopram (10 mg/day starting dose, after a week 20 mg/day). Group 3: healthy control.	Blood test, and familiar report for alcohol use, BDI, AUQ-K, CGI, MAST	AUQ-K: significantly reduced in group 1 *p* = 0.02. AUQ-K ns reduction in group 2 group or in group 3. AUQ-k significant reduction in group 1 *vs.* group 2. Ns difference in alcohol-free period between the 3 groups.	BDI and CGI: significant reduction in both group 1 and Group 2 *vs.* group 3. Ns differences between group 1 *vs.* group 2 in BDI and CGI.
Sulaiman *et al.*, 2013 [30] RCT	N = 37, N*_m_* = 35; Group 1 N = 19, N*_m_* = 18; Group 2 N = 18, N*_m_* = 17; Methamphetamine dependence DSM 4 history of psychosis	Group 1: aripiprazole 5-10 mg/day. Group 2: placebo	MINI, BSCS, PANSS, CGI, AIMS, SAS, BAS	Group 1 *vs.* Group 2 ns difference in the maintenance of abstinence. Group 1 *vs.* Group 2 Significantly more days spent in treatment and Significant craving reduction in BSCS.	PANSS significant decrease Group 1 *vs.* Group 2. CGI significant decrease in Group 1 *vs.* Group 2.
Skryabin *et al.*, 2021 [31] RCT	N = 90, N*_m_* = 90; Group 1: N = 30, N*_m_* = 30 Group 2: N = 30, N*_m_* = 30 Group 3: N = 30, N*_m_* = 30 Schizophrenia spectrum disorders (ICD-10). Mental and behavioral disorders due to multiple drug use (ICD-10).	Group 1: aripiprazole up to 20 mg/day (minimal dose 10 mg/day). Group 2: quetiapine up to 600 mg/day (minimal dose 300 mg/day). Group 3: haloperidol: up to 30 mg/daily (minimal dose 15 mg/day).	BPRS; PANSS; VAScrav; SCS; ASI the Naranjo Algorithm	Group 1 *vs.* group 3 is significantly more effective in the reduction of craving. Group 1 *vs.* group 2 is significantly more effective in the reduction of craving.	Group 1 *vs.* group 3 was significantly more effective in the reduction of PANSS and BPRS. Group 1 *vs.* group 2 was significantly more effective on PANSS general symptoms.

**Abbreviations**: AIMS: Abnormal Involuntary Movement Scale; ASI: Addiction Severity Index; AUQ-K: Korean Alcohol Urge Questionnaire; BAS: Barnes Akathisia Scale; BDI: Back Depression Inventory Scale; BPRS: Brief Psychiatric Rating Scale; BSCS: Brief Substance Craving Scale; CGI: Clinical Global Impression; CIWA-ar: Clinical Institute Withdrawal Assessment for Alcohol; DOTES: Dosage Record Treatment Emergent Symptom Scale; EuropASI: European version of the Addiction Severity Index; HAM-A: Hamilton Rating Scale for Anxiety; HAM-D: Hamilton Rating Scale for Depression; MADRS: Montgomery-Asberg Depression Rating Scale; MAST: Michigan alcohol screening test; MINI: Mini-International Neuropsychiatric Interview; MSES: Medical Side Effect Summary; OCDS: Obsessive and Compulsive Drinking Scale; PANNS: Positive and Negative Symptom Scale; QOL index: the Quality of Life Index; SANS: Scale for Assessment of Negative Symptoms; SAPS: Scale for Assessment of Positive Symptoms; SAS: Simpson Angus Scale; SCID I and II: Structured Clinical Interview for DSM-IV ; SCL-90 R: Symptom Check List 90 Revised; SCS: Substance Craving Scale; SDS: Severity of Dependence Scale; TLFB: Time Line Follow-Back; TLFDA: the Time Line Follow-Back for Drugs and Alcohol; VAScrav: Visual Analogue Scale for substance Craving; WHODAS-2.0: World Health Organization Disability Assessment Schedule; WHOQOL-BREF: World Health Organization Quality-of-Life Scale; YMRS: Young Mania Rating Scale.

## LIST OF ABBREVIATIONS

BDI: Depression Inventory Scale
BPRS: Brief Psychiatric Rating Scale
cAMP: cyclic adenosine monophosphate
CGI: Clinical Global Impression
MDD: Major Depressive Disorder
PANSS: Positive and Negative Symptom Scale
RCT: Randomized Controlled Trials
SUD: Substance Use Disorder
